# Azelaic acid-integrated therapeutic deep eutectic systems: overcoming solubility and permeability barriers for enhanced transdermal drug delivery

**DOI:** 10.1039/d5ra09988a

**Published:** 2026-04-13

**Authors:** Liyong Du, Shixian Wang, Haihui Chen, Chunhui Liu, Shuyan Yang, Yue Wang, Beilei Cai, Jingguo Yang, Yuqiang Ding

**Affiliations:** a Key Laboratory of Synthetic and Biological Colloids, Ministry of Education, School of Chemical and Material Engineering, Jiangnan University Wuxi 214122 China dlyong@jiangnan.edu.cn; b Key Laboratory of Micro-Nano Materials for Energy Storage and Conversion of Henan Province, Institute of Surface Micro and Nano Materials, College of Chemical and Materials Engineering, Xuchang University Xuchang Henan 461000 China; c Wuxi Zhiyan Biotechnology Co., Ltd Xuxi 214194 China

## Abstract

Azelaic acid (AzA) is a saturated dicarboxylic acid used to treat skin disorders like acne, rosacea, and melasma. However, its transdermal application is limited by its poor water solubility and permeability. In this study, therapeutic deep eutectic systems (THEDES) are synthesized by combining azelaic acid (AzA) and D-panthenol (DP) in various molar ratios (3 : 1, 2 : 1, 1 : 1, 1 : 2, 1 : 3). The optimal molar ratio of the THEDES (AzA : DP = 1 : 2) is analyzed with molecular simulation calculations, polarized optical microscopy (POM), Fourier Infrared Spectroscopy (FTIR), Nuclear Magnetic Resonance (1H NMR), Thermogravimetric Analysis (TGA), and water solubility and stability tests. In addition, the THEDES system is evaluated for toxicity, antibacterial and anti-inflammatory efficacy, and transdermal properties. The results show that it outperforms AzA raw material by demonstrating good water solubility and permeability, lower transdermal toxicity and skin irritation, and improved bioactivity compared to AzA raw material. Furthermore, its antimicrobial, anti-inflammatory, and transdermal properties are also superior to those of the AzA raw material. Using molecular docking analysis and molecular dynamics simulation, its mechanisms of action in the treatment of acne and skin permeation are investigated. In conclusion, AzA-DP THEDES effectively resolves AzA's solubility and permeability issues while enhancing its efficacy in Transdermal Drug Delivery Systems (TDDS).

## Abstract 2

Azelaic acid (AzA) is a saturated dicarboxylic acid used to treat skin disorders like acne, rosacea, and melasma.

## Graphical abstract

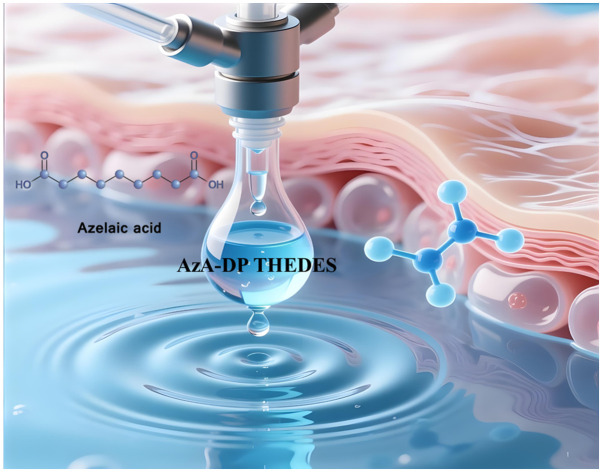

## Introduction

Acne is a cutaneous inflammatory disorder of the hair follicles and their accompanying sebaceous glands, occurring primarily on the face and trunk. It affects around 9% of the global population, with about 85% of those affected being individuals aged between 12 and 29 years.^1,2^ Acne has the potential to result in lasting physical scars, contribute to considerable psychological distress such as anxiety and depression, adversely affect self-esteem and overall quality of life, and is linked to increased suicidal ideation.^3,4^ Azelaic acid (AzA) is a saturated dicarboxylic acid that occurs naturally in grains like rye, barley, and wheat, and it also be produced from yeast.^5^ AzA reduces acne proliferation, aids in the normalization of keratin production, and functions as a direct anti-inflammatory agent by inhibiting the production of hydroxyl and superoxide radicals by neutrophils.^6^ It demonstrates remarkable therapeutic efficacy in treating acne vulgaris and inflammatory acne (subcutaneous pustules, nodules, and nodular cystic acne) when applied topically, usually as a 15–20% cream).^7^

Transdermal drug delivery systems (TDDS) have become the third most common route of administration after injections and oral administration in recent decades.^8^ However, the development of effective TDDS for various active ingredients remains a major challenge. Approximately 40% of the oral medications available are reported to exhibit poor solubility and permeability because of the skin's impermeability, making them ineffective for administration through transdermal and posterior systems.^9^ These limitations are similarly observed with AzA, which demonstrates limited solubility in water (∼0.24 g/100 g water at 25 °C), and at this concentration, it fails to provide effective treatment.^10^ Consequently, higher concentrations of AzA are needed for strong clinical results; however, this often leads to skin irritation, burning, and stinging, making the formulation poorly tolerated and unsuitable for sensitive skin. Furthermore, in formulations with high concentrations of AzA, it tends to crystallize and precipitate easily, leading to limited transdermal efficiency and bioavailability.^11^

Currently, researchers have developed a number of strategies to address the previously mentioned limitations of AzA to achieve better therapeutic outcomes. These strategies include microemulsions, liposomes, hydrogels, and cyclodextrin encapsulation.^12–16^ However, these traditional strategies have inherent limitations for AzA delivery: microemulsions require high concentrations of surfactants that easily cause skin irritation; liposomes and hydrogels suffer from drug leakage, crystallization and poor transdermal efficiency; cyclodextrin encapsulation only improves solubility with negligible permeation enhancement effect. In contrast, therapeutic deep eutectic systems (THEDES) show unique advantages over the above strategies. As supramolecular systems formed by active pharmaceutical ingredients (APIs) and biocompatible ligands *via* hydrogen bonds, THEDES feature a carrier-free design to avoid excipient-related irritation, simultaneously break the solubility and permeability barriers of poorly soluble drugs, and exhibit excellent anti-crystallization stability with a simple, solvent-free and scalable preparation process. These advantages make THEDES a promising strategy to address the delivery limitations of AzA. Therapeutic deep eutectic systems (THEDES) are supramolecular systems formed by two or more components that interact through non-covalent bonding forces such as hydrogen bonds. One of these components is an API, while the other acts as a hydrogen bond donor (HBD) or a hydrogen bond acceptor (HBA).^17^ For example, aspirin can act as an HBD to form deep eutectic system (DES) with an HBA like with choline chloride,^18^ and lidocaine can be an HBA that forms DES a carboxylic acid (which acts as the HBD).^19^ In addition to their fundamental properties such as low vapor pressure, high tunability, and electrical conductivity, these types of DES exhibit excellent solubility, permeability, and absorption of APIs. This may lead to enhanced biocompatibility and biodegradability compared to traditional DES.^20^ In one report, the membrane permeability of ibuprofen (IBF) with L-menthol or DL-menthol after the formation of a THEDES system was 2-fold and 3-fold higher than that of the corresponding ibuprofen (IBF) powder, respectively.^21^

Deep eutectic solvents (DESs) are defined as binary/multi-component homogeneous mixtures formed by specific hydrogen-bond interactions between hydrogen bond donors (HBDs) and hydrogen bond acceptors (HBAs). The core thermodynamic feature that distinguishes genuine DES from simple physical mixtures is significant deviation from ideal mixing behavior, accompanied by a markedly reduced phase transition temperature (melting point for crystalline DES, glass transition temperature *T*_g_ for amorphous DES) compared with individual pure components. For amorphous DES systems with inhibited crystallization, the non-ideality can be systematically verified *via* thermodynamic modeling of composition-dependent *T*_g_ data, which is the widely accepted standard for thermodynamic substantiation of DES formation.

In the present work, D-panthenol (vitaminogen B5),^22^ another naturally occurring substance that is mostly present in foods like yeast, liver, eggs, and milk, was chosen as a ligand to form stabilized THEDES with AzA. This active system is capable of effectively addressing the issues related to the low water solubility and inadequate transdermal characteristics of AzA, thereby allowing it to serve as a direct raw material for innovative AzA active skin delivery pharmaceuticals. In this study, the optimal molar ratio was established through molecular simulation. The intermolecular interactions and the mechanism of formation were examined using FTIR and 1H NMR. The thermal stability of the system was determined by TGA, and the solubility and stability experiments verified its water solubility and stability. In addition, the toxicity, bacteriostatic, anti-inflammatory efficacy, and permeability of AzA-DP THEDES were systematically investigated in and compared with AzA. Lastly, the bioactivity of AzA-DP THEDES was assessed in a clinical efficacy study. The mechanisms of action in acne treatment and skin penetration were explored through molecular docking and molecular dynamics simulations.

## Results and discussion

### Formation of THEDES, solubility and stability studies

**Fig. 1:**
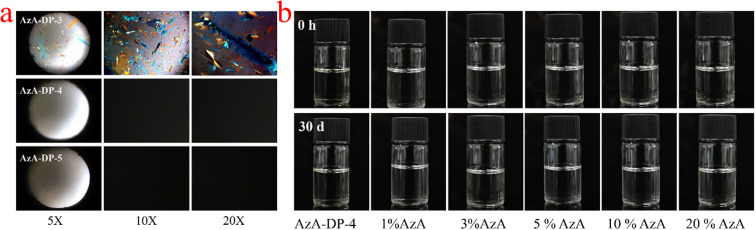
(a) POM images of AzA-DP-3, AzA-DP-4, and AzA-DP-5 (b) water solubility and stability of AzA-DP-4.

THEDES are formed by specific intermolecular interactions between the API and one or more ligands. The molar ratio of API to ligand is a crucial factor in determining whether a stable THEDES will form, and it influences the system's properties.^23^ Five types of mixtures of AzA : DP were prepared according to different molar ratios (3 : 1, 2 : 1, 1 : 1, 1 : 2, 1 : 3) (defined sequentially as AzA-DP-1, AzA-DP-2, AzA-DP-3, AzA-DP-4, AzA-DP-5) to determine the appropriate formulation for the synthesis of THEDES. The formation of the liquid mixtures was observed directly and documented through microscopic images. Table 1 summarizes the states of the systems formed at different molar ratios and their appearance and properties after 24 h. AzA-DP-3, AzA-DP-4, and AzA-DP-5 were homogeneous and transparent liquids that were observable with the naked eye and remained stable without recrystallisation on cooling, whereas AzA-DP-1 and AzA-DP-2 appeared as solid–liquid mixtures at room temperature. The samples (AzA-DP-3, AzA-DP-4, and AzA-DP-5) that could form a liquid system were analyzed using polarized light microscopy after being maintained at room temperature for 24 h. The results are shown in Fig. 1a. Although AzA-DP-3 can also form a liquid system visible to the naked eye upon heating, a few crystals can still be observed using polarization microscopy. In contrast, AzA-DP-4 and AzA-DP-5 maintain homogeneous and transparent systems, with no crystal precipitation observed. In general, molecules in the THEDES interact through an extensive network of hydrogen bonds, which establishes a stable, balanced intermolecular force that prevents crystallization.^24^ However, weak bonds or interactions between the two components lead to crystallization due to an unstable system. From the above results, AzA-DP-4 was observed to form a stable system. While AzA-DP-5 also formed a liquid-stabilized system, it contained an excess of DP. To achieve a high loading of azelaic acid, AzA-DP-4 was selected for further studies and characterization.

**Table 1:** State of the system with different molar ratios

Sample name	AzA : DP	Heated state	System status after 24 h
AzA-DP-1	3 : 1	Solid–liquid mixture	Solid–liquid mixture
AzA-DP-2	2 : 1	Solid–liquid mixture	Solid–liquid mixture
AzA-DP-3	1 : 1	Liquid	Solid–liquid mixture
AzA-DP-4	1 : 2	Liquid	Liquid
AzA-DP-5	1 : 3	Liquid	Liquid

Water is a preferred solvent; however, THEDES are stabilized by an extensive network of strong hydrogen bonds, and water disrupts this network, altering the system's stability. Fig. 1b shows the characteristics of AzA-DP-4 and its aqueous solutions with different mass fractions (1%, 3%, 5%, 10%, and 20% AzA, respectively) following 30 d at room temperature. Neither the AzA-DP-4 system nor its various concentrations of aqueous solutions resulted in any delamination phenomenon or precipitation following the 30 d. To further evaluate the physical stability and recrystallization tendency of the optimized THEDES under stressed conditions, accelerated stability studies were conducted. Aqueous solutions of AzA-DP-4 with different AzA concentrations (1%, 5%, 10%, and 20%, w/w) were stored at 45 °C for 30 days. As shown in Fig. S1 (SI), all tested solutions maintained their homogeneous and transparent appearance throughout the 30-day period, with no evidence of turbidity, precipitation, or recrystallization. The systems remained as homogeneous and transparent liquid systems, indicating that the AzA-DP-4 system has good water solubility and stability.

### Computational analysis of Gaussian molecular simulations

**Fig. 2:**
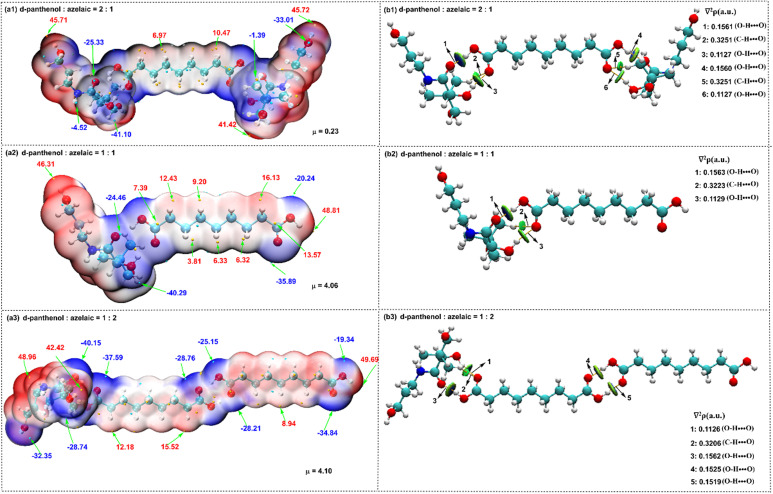
Gaussian calculations of AzA and DP with different molar ratio (a) ESP; (b) NCI and AIM analysis.

Fig. 2 shows the electrostatic potential (ESP) and surface extremes for different ratios of DP and AzA. The local surface minima and maxima of the ESP are indicated by green and orange spheres, respectively. The structure depicted in Fig. 2(a1) (DP : AzA = 2 : 1) exhibits a concentrated electrostatic potential (ESP) that is nearly zero, characterized by the smallest difference between its maximum and minimum values. Additionally, the associated dipole moment was low (*µ* = 0.23D), contributing to the reduced polarity and enhanced stability of this structure. In addition, qualitative non-covalent interaction (NCI) analysis and quantitative atom-in-molecule (AIM) analysis were performed to distinguish the types and strengths of weak interactions in different proportions of DP and AzA (Fig. 2b).

The large blue and green areas between the ions indicate abundant hydrogen bonding and van der Waals interactions, respectively. The orange spheres are the bond critical points (BCPs) of AIM, whose properties quantitatively describe the nature and strength of the interactions. The strength of these interactions can be predicted from the Laplace value of the electron density (∇2*ρ*). When the Laplace values of the electron density (∇2*ρ*) were compared across the three ratios, it is observed that the strongest weak interaction occurred for the DP : AzA molar ratio of 2 : 1 in the structure of AzA-DP-4. This suggests that both strong and weak interactions contribute positively to the stability of the structure.

### Characterization analysis

**Fig. 3:**
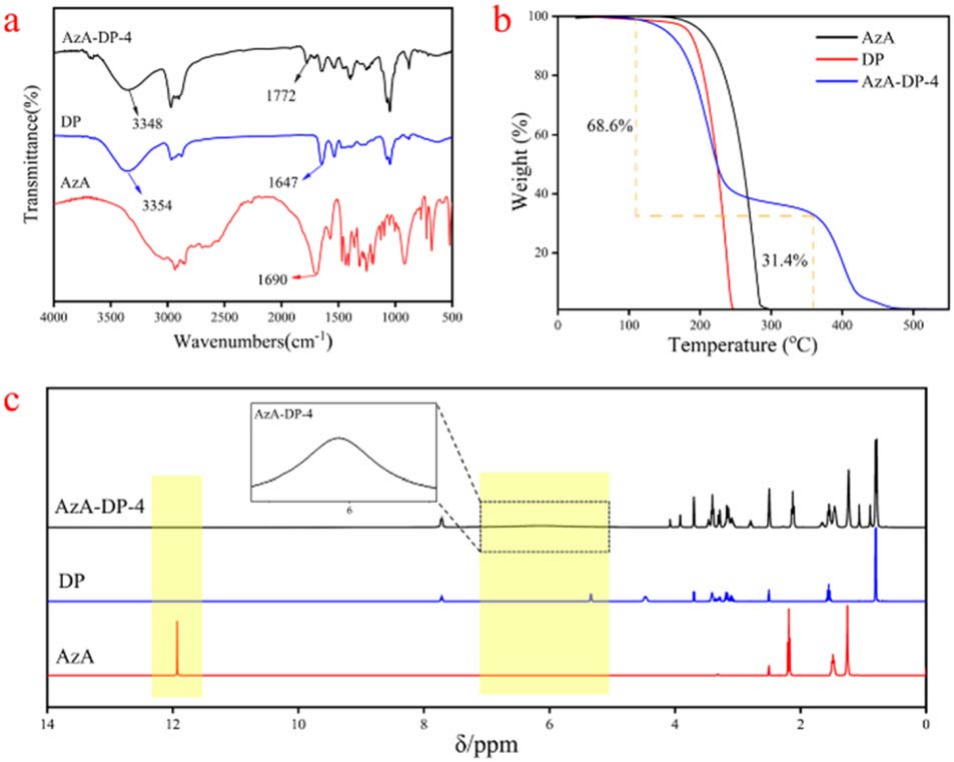
Characterization of AzA, DP and THEDES (a) FTIR (b) TGA (c) 1H NMR.

The FTIR spectra of the THEDES (AzA-DP-4) system and its individual single-component components are shown in Fig. 3a. AzA is a long straight-chain dicarboxylic acid, with a carbonyl peak at 1690 cm^−1^. The peaks observed in DP within the range of 3200 to 3600 cm^−1^ were due to the telescopic vibrations of –OH and N–H groups, while the peak at 1647 cm^−1^ was associated with the C=O bond. The –OH peak in the THEDES (AzA-DP-4) system experienced a shift from 3354 cm^−1^ to 3348 cm^−1^ when compared to DP. Additionally, the C=O peak position also shifted from 1690 cm^−1^ and 1647 cm^−1^ to 1772 cm^−1^ in comparison to the AzA and DP feedstock fractions, indicating a significant shift towards higher wave numbers. The significant displacement was attributed to the strong intermolecular interactions between the carboxylic acid groups in the AzA molecule and the hydroxyl and carbonyl groups in DP, which resulted in the formation of a large number of hydrogen bonds. A similar phenomenon was observed in the THEDES system prepared by other researchers.^25^

Fig. 3b shows the dynamic TGA curves of AzA-DP-4 and its component feedstocks, represented as a percentage of weight loss. AzA-DP-4 demonstrated two stages of degradation with the first weight loss of 68.6% and the second weight loss of 31.4%. These data are in line with the constitution of AzA-DP-4 (molar ratio of AzA : DP = 1 : 2). In addition, corresponding to DP, the initial decomposition of AzA-DP-4 also began at around 130 °C. The second stage of decomposition, however, occurred above 300 °C, illustrating the higher decomposition temperature of AzA because of the strong interaction forces in AzA-DP-4. The TGA test showed that the strong intermolecular interaction bonds contributed to the thermal stability of the prepared AzA-DP-4.

The DTG curve (Fig. S2) shows that neat AzA and neat DP exhibited a single mass-loss peak with Tmax values at 230 °C and 280 °C, respectively. In contrast, AzA-DP-4 displayed two distinct mass-loss steps. The first mass-loss peak (*T*_max_ = 220 °C) was attributed to the decomposition of the DP component, with an onset decomposition temperature (132 °C) slightly higher than that of neat DP (130 °C). The second mass-loss peak (*T*_max_ = 390 °C) corresponded to the decomposition of the AzA component, whose decomposition temperature was significantly elevated compared with that of neat AzA (280 °C). This phenomenon can be ascribed to the synergistic effect of the hydrogen-bonding network formed upon THEDES fabrication. The mass ratio of the two decomposition stages (68.6%/31.4%) corresponded precisely to the mass fractions of DP and AzA, which was consistent with the theoretical stoichiometry of a 1 : 2 molar ratio, confirming the stoichiometric integrity of the THEDES.

^1^H NMR provided the chemical environments of hydrogen atoms in the THEDES (AzA-DP-4) system. As shown in Fig. 3c, the carboxyl active hydrogens of AzA were found at 11.93 ppm and the hydroxyl active hydrogen (–CH(OH)CONH–) of DP was observed at 5.36 ppm. In comparison to these peaks, the hydrogen proton of AzA-DP-4 exhibited a broad peak ranging from 4.8 to 7.3 ppm, which attributes to the hydrogen-bond interactions among carbonyl and hydroxyl (–CH(OH)CONH–) groups from each other.^26^ The nitrogen–hydrogen bond peak (–CH(OH)CONH–) of DP at 7.72 ppm had not undergone any significant chemical shift when compared to AzA-DP-4, indicating the absence of hydrogen-bond interactions from this group. All of these data are consistent with the computational simulation above (Fig. 2). In addition, by integrating the peaks at the other positions, it was determined that the two-component raw material formed the THEDES system at a molar ratio of AzA : DP = 1 : 2.

### Thermodynamic characterization of DES *via* DSC

The glass transition temperature (*T*_g_) is a key parameter to verify the homogeneity and intermolecular interactions of the deep eutectic system. As shown in Fig. S4, all AzA-DP mixtures exhibited a single, well-defined glass transition step, with no crystallization or melting peaks detected in the scan range from −100 °C to 50 °C. This confirms that all mixtures are homogeneous, single-phase amorphous undercooled liquids, with no phase separation or recrystallization tendency. The *T*_g_ of the system decreased monotonically with the increase of azelaic acid (AzA) mass fraction, from −32.17 °C (pure D-panthenol, DP) to −49.17 °C (AzA : DP = 1 : 1). This trend is attributed to the plasticizing effect of small-molecule AzA: the specific hydrogen–bond interaction between AzA and DP breaks the self-association hydrogen network of pure DP, improving the molecular mobility of the system, thus reducing the *T*_g_ of the mixture. To quantitatively evaluate the intermolecular interaction between AzA and DP, the Gordon–Taylor equation was used to fit the experimental *T*_g_ data. The fitting results showed that the interaction parameter *k* = 0.72, which significantly deviated from 1, confirming the strong non-ideal mixing and specific hydrogen–bond interaction between AzA and DP. Notably, the optimal mixture (AzA : DP = 1 : 2) showed the smallest residual between the experimental and fitted *T*_g_ values, indicating the highest degree of non-ideal mixing and the strongest intermolecular interaction at this molar ratio, which is consistent with its optimal solubilization, skin permeation and bioactivity performance. To further demonstrate the non-ideal mixing behavior of the AzA-DP system, the Flory–Huggins interaction parameter *χ* was calculated based on the Gordon–Taylor fitting results. The obtained *χ* = −0.83 (significantly less than 0 for ideal mixing) confirms the strong specific hydrogen–bond interaction between the carboxyl group of AzA and the hydroxyl group of DP, which is the intrinsic driving force for the formation of the DES system.

To eliminate the interference of trace moisture on the thermodynamic properties of the system, the moisture content of all formulations was quantitatively determined using the Karl Fischer titration method. The results showed that the moisture content of all formulations was 1.69 wt%. This extremely low moisture content was insufficient to produce a significant plasticizing effect on the glass transition temperature of the system. Moreover, the *T*_g_ of the system showed a monotonic and regular change with the increase of the mole fraction of AzA, without any abnormal jumps, proving that the change in *T*_g_ originated from the specific hydrogen bond interaction between AzA and DP, rather than the interference of moisture. At the same time, all formulations presented a single, non-split *T*_g_ step, indicating that the system was a homogeneous binary single-phase DES without forming a DP-AzA-water ternary system.

The specific hydrogen–bond interaction between azelaic acid (AzA, hydrogen bond donor, HBD) and D-panthenol (DP, hydrogen bond acceptor, HBA) is the intrinsic driving force for DES formation, and the absence of esterification side reaction is the prerequisite for confirming the physical nature of the DES system. The ^13^C NMR spectra of the optimal AzA-DP DES (1 : 2 molar ratio) were characterized (S5).The esterification reaction between the carboxyl group of AzA and the hydroxyl group of DP requires harsh conditions (strong acid catalyst, high temperature >120 °C, continuous dehydration), while our DES was prepared under mild conditions (70 °C, atmospheric pressure, no catalyst, no dehydration), which does not support the occurrence of esterification. The ^13^C NMR results further provide direct and sufficient evidence to exclude esterification: no new ester carbonyl peak: the characteristic peak of aliphatic ester carbonyl carbon is in the range of 172–174 ppm. No new characteristic peak was detected in this range in the DES spectrum, except for the inherent amide carbonyl peak of DP (171.8 ppm), which rules out the formation of ester bonds.

No shift of hydroxyl-linked carbon peaks: if esterification occurs, the hydroxyl-linked methylene carbon of DP (–CH_2_OH) will be converted to ester-linked methylene carbon (–CH_2_OOC–), and its chemical shift will show a significant low-field shift of 5–7 ppm. However, no significant shift of these peaks was observed in the DES spectrum, which further confirms that no esterification reaction occurs.

### Thermodynamic analysis

#### Thermodynamic substantiation of DES formation

The core thermodynamic criterion for DES formation is significant deviation from ideal mixing, driven by specific hydrogen-bond interactions between HBD and HBA. Based on the DSC-measured composition-dependent *T*_g_ data, we systematically verified the non-ideality of the AzA-DP system *via* ideal mixing comparison, thermodynamic modeling, and activity coefficient estimation.

#### Comparison with ideal mixing theory

The measured *T*_g_ of all AzA-DP formulations was compared with the ideal mixing *T*_g_ calculated *via* the Fox equation (Table S6). All tested formulations showed a significant deviation between the measured *T*_g_ and the ideal *T*_g_, which directly proves that the system is not an ideal physical mixture. The optimal 1 : 2 formulation exhibited the most significant deviation, indicating the strongest non-ideal intermolecular interaction at this molar ratio.The composition-dependent *T*_g_ profile is further visualized in Fig. S7. The measured *T*_g_ curve showed a clear deviation from the ideal mixing line predicted by the Fox equation, which provides intuitive evidence of the non-ideal mixing behavior of the system.

### Thermodynamic modeling *via* Gordon–Taylor model

The experimental *T*_g_ data was fitted *via* the Gordon–Taylor model, with a high goodness of fit (*R*^2^ = 0.9992), confirming that the model is fully applicable to the AzA-DP system. The core fitting parameters are summarized in Table S8. The interaction parameter *k* = 0.72 significantly deviates from 1, which quantitatively confirms the strong specific hydrogen–bond interaction between the carboxyl group of AzA and the hydroxyl group of DP. This is the intrinsic driving force for DES formation, and is fully consistent with the ^13^C NMR characterization results (the significant chemical shift of the carboxyl carbon of AzA in the DES system).

### Activity coefficient estimation and verification

To further quantify the deviation from ideal solution behavior, the Flory–Huggins interaction parameter and the activity coefficient of AzA in the optimal 1 : 2 formulation were calculated. The Flory–Huggins interaction parameter *χ* = −0.83 (significantly less than 0 for ideal mixing), which further confirms the strong attractive hydrogen–bond interaction between AzA and DP.

The activity coefficient of AzA in the optimal DES formulation was calculated to be *γ* ≈ 0.68, which is significantly less than 1 (the value for ideal solutions). This result quantitatively confirms that the system deviates markedly from ideal mixing behavior, and is fully consistent with our isoconcentration control experiments: under the same total AzA concentration, the skin permeation efficiency and biological activity of the DES group were significantly higher than those of the control group, which directly proves that the effective thermodynamic activity of AzA in the DES system is significantly enhanced.

The strong hydrogen–bond interaction between AzA and DP effectively inhibits the self-aggregation and crystallization of AzA, reduces its fugacity coefficient, and thus improves its effective thermodynamic activity. This is the core mechanism for the performance enhancement of the DES system, and also provides sufficient thermodynamic substantiation for the definition of the deep eutectic system.

Based on the above characterization results, the AzA-DP system prepared in this study can be clearly distinguished from the co-amorphous system and the simple solubilization system in terms of their fundamental nature: firstly, this system is a uniformly clear and flowable liquid at room temperature, with no crystallization/melting characteristic peaks in the full temperature range DSC scan, and no crystallization or turbidity during a one-month accelerated stability test, which is fundamentally different from the co-amorphous system that is an unstable solid at room temperature and has an inherent tendency to crystallize; Secondly, the ^13^C NMR results confirmed the existence of specific hydrogen bond interactions between AzA and DP, and the thermodynamic fitting results showed that the system significantly deviated from the ideal mixed behavior, and the equal concentration control experiments also proved that its performance improvement was not due to the simple solute solubilization effect, which is completely different from the ideal solubilization system without specific intermolecular interactions.

### Dermal toxicity test

The acute percutaneous (dermal) toxicity test results indicate that the test substance, AzA-DP-4, did not cause any poisoning in the rats. The results showed no poisoning or mortality in the rats over the 14-d observation period. At the end of the observation period, the animals underwent gross necropsy, and no abnormal changes were observed in the major organs by naked eye. Changes in body weight and deaths of the animals are shown in Table 2. The results of the tests showed that the acute percutaneous LD_50_ of AzA-DP-4 for rats was 2180 mg kg^−1^ body weight, which is slightly toxic. According to the Globally Harmonized System of Classification and Labelling of Chemicals (GHS, Rev.9, 2021) and the Chinese National Standard GB 30000.18-2013, substances with an acute dermal LD_50_ value within the range of 2000–5000 mg kg^−1^ body weight are classified as Category 5, corresponding to the description of “slight toxicity”. In the present study, the measured acute dermal LD_50_ value of AzA-DP-4 was 2180 mg kg^−1^, which falls within the above range. Furthermore, all experimental animals in Table 2 showed no mortality during the 14-day observation period and exhibited normal body weight gain, further supporting the absence of obvious lethal or systemic toxicity at the tested doses.

**Table 2:** Changes in body weights and deaths of experimental rats^a^

Sex of animals	Drug dose (mg kg^−1^)	Number of experimental animals	Weight (*X* ± SD)			Number of deaths	Mortality rate (%)
			0 d	7 d	14 d		
Males	2180	5	236.5 ± 3.2	278.6 ± 4.3	334.2 ± 4.1	0	0
Females	2180	5	233.4 ± 2.3	256.5 ± 4.7	281.1 ± 5.8	0	0

a Note: according to the GHS classification system, substances with a transdermal LD_50_ value in the range of 2000–5000 mg kg^−1^ are categorized as Class 5 (slight toxicity). In the present study, the transdermal LD_50_ value was determined to be 2180 mg kg^−1^, which falls within this range; therefore, the test substance is classified as slightly toxic.

### Irritation test

In the skin irritation test performed on New Zealand rabbits, no redness or inflammation of the skin of the test animals was observed. In addition, the test animals did not show any signs of discomfort. The results of the skin irritation/corrosion test performed after 14 days showed that both the daily mean and total values were 0 (Table S1). In the acute eye irritation test performed on New Zealand rabbits, test animals were observed for signs of eye irritation, particularly of the cornea, iris, and conjunctiva. No signs of redness, swelling, or congestion of the eyes of the test animals were observed. The test animals exhibited no changes in mobility, and there were no indications of intoxication or death during the test. In addition, there was no rinsing throughout. The scoring was determined using the acute eye irritation/corrosivity scale, which evaluates the maximum average irritation responses of the animals' cornea, iris, and conjunctiva after applying the samples at the specified observation intervals of 24, 48, and 72 hours, as well as during the following recovery period. The results of the test were 0 for all animals (Table S2), which indicates that AzA-DP-4 had a very low level of ocular irritation.

The results of the irritation experiments showed that no adverse reaction or irritation was observed in all test animals, and no lethality was observed. It can be concluded that AzA-DP-4 has extremely low irritation and is safe for use.

### Permeability analysis

Under the dilution conditions used in this biological test, the retention and mechanism of action of the core structure of DES were verified in the following two aspects: firstly, ^13^C NMR and thermodynamic fitting results confirmed that the specific hydrogen bond binding energy between AzA and DP was significantly higher than that between the components and the diluent solvents (water, propylene glycol, DMSO), and the core hydrogen bond network of DES could be stably retained under a fixed dilution ratio; at the same time, all biological tests set strict equal concentration control groups (with the total AzA concentration of the DES group being completely consistent), the transdermal efficiency and biological activity of the DES group were still significantly superior to those of the control group, directly confirming the retention of the DES structure. Secondly, based on the Flory–Huggins model, the activity coefficient of AzA in DES was calculated to be approximately 0.68 (significantly less than 1 of an ideal solution), quantitatively proving that DES could significantly enhance the effective thermodynamic activity of AzA. The equal concentration control experiments completely excluded the interference of concentration differences, confirming that the performance improvement originated from the improvement of the thermodynamic activity of AzA by DES, supporting the mechanism of this study.

**Fig. 4:**
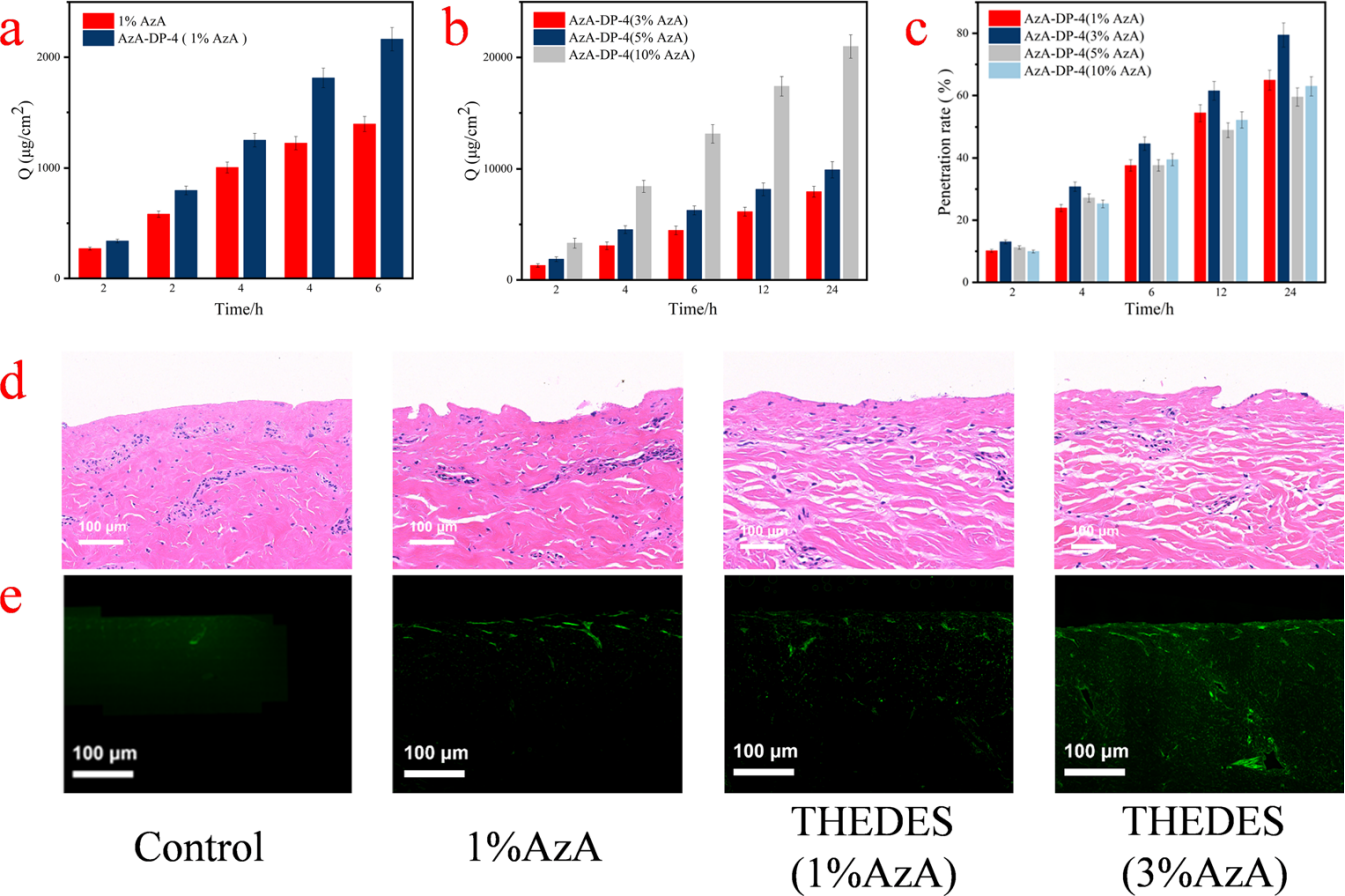
Cumulative permeation per unit area of (a) THEDES and AzA group at the same concentration (1%AzA) (b) THEDES at different concentrations (3%, 5%, 10%AzA) (c) permeation rate of THEDES at different concentrations (1%, 3%, 5%, 10%AzA) (d) H&E staining image of skin (e) fluorescence image.

The permeation effect of the aqueous AzA-DP-4 was evaluated at various concentrations (1%, 3%, 5%, and 10%, based on the content of AzA), along with AzA as a comparison. The concentration of AzA in the comparison group was set as 1% and propylene glycol was chosen as the solvent to achieve dissolution. The findings are illustrated in Fig. 4a. The 24 h cumulative permeation amount per unit area of AzA (1%) was 1398.47 µg cm^−2^. At the same concentration of AzA, the 24 h cumulative permeation amount per unit area of AzA-DP-4 was 2163.33 µg cm^−2^, which is 1.5 times greater. In addition, Fig. 4b and c showed that the permeation performance of the THEDES system is related to drug concentration. At an AzA concentration of 10%, AzA-DP-4 exhibited the highest 24 h cumulative permeation amount per unit area of 20 980.88 µg cm^−2^. Nevertheless, AzA-DP-4 achieved the highest 24-h permeation rate of 39.72% with the AzA concentration of 3%, when comprehensively considering the dosage administration. This indicates that intermolecular forces in AzA-DP-4 may open the tight junctions of the skin stratum corneum, weakening the strength of the lipid network on the skin surface, and promoting the penetration.^27^ However, an excessively high concentration may result in issues such as an increase in the system's viscosity, thereby impacting its permeability.^28^

Fig. 4d shows the H&E staining images of the skin after the transdermal experiment, and changes in the structure and morphology of the skin stratum corneum can be observed. The loose and disorganized stratum corneum in the AzA raw material group illustrated more damage. Comparatively, the stratum corneum treated by AzA-DP-4 exhibited no significant changes and remained tight. This suggests that AzA-DP-4 caused minimal damage to the skin. Furthermore, it was observed that the cellular interstitial space of the skin increased following the treatment of AzA-DP-4 with 3% AzA, which is more conducive for drug penetration. Fig. 4e shows the fluorescence analysis. As seen, the fluorescent dyes in the blank control, AzA raw material groups were primarily located in the skin's surface layer, facing difficulties in permeation and exhibiting uneven distribution. The THEDES (AzA-DP-4) system, however, had a uniform distribution and penetrated deeper into the skin. In 3% AzA-DP-4 group, it was particularly observed that the skin fluorescence intensity was higher, which was consistent with the results of the *in vitro* percutaneous permeation test.

All above results demonstrate that THEDES can exhibit excellent permeability without damaging the skin barrier, significantly enhancing the transdermal delivery capability of AzA.

### Molecular dynamics simulation of skin permeation

**Fig. 5:**
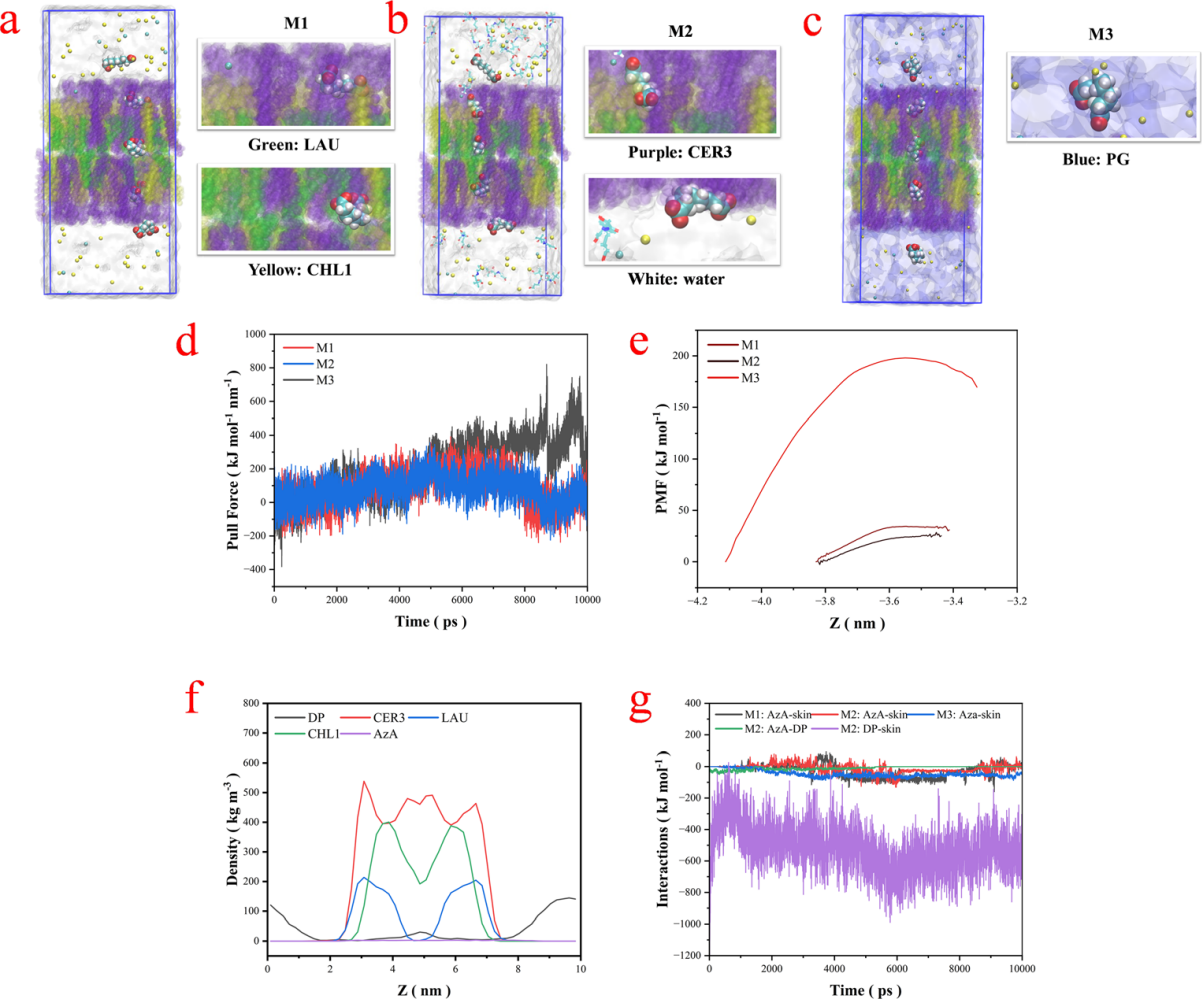
(a)–(c) Transdermal permeation processes of AzA in M1, M2 and M3. (d) Pulling forces required to pull AzA across the skin barrier model in M1, M2 and M3. (e) PMF for the transmembrane process of AzA in M1, M2 and M3. (f) Density distributions of each molecule along the *Z*-axis (permeation direction) in M2. (g) Total interaction energies between different components in M1, M2 and M3.

The mechanism of THEDES-mediated permeation enhancement remains challenging to characterize experimentally due to its complex physical nature. This study employed molecular dynamics (MD) simulations to elucidate the transdermal permeation process of supramolecular azelaic acid (AzA). A simplified stratum corneum (SC) lipid matrix model was constructed as a bilayer containing ceramide (CER3), cholesterol (CHL1), and lauric acid (LAU) in a 1 : 1 : 1 molar ratio.^29,30^ Three systems were simulated: AzA permeation from pure water (M1) and from an aqueous deep eutectic solvent (DP) solution (M2) and from a Propylene glycol solvent solution (M3). The potential of mean force (PMF) derived from umbrella sampling simulations quantified the pulling forces required for AzA penetration through the skin barrier. Fig. 5a–c visualize the AzA permeation pathway through sequential simulation snapshots. Comparative force analysis reveals a 17.6% reduction in the required pulling force for AzA permeation in THEDES-containing system M2 (28.5 kJ mol^−1^) *versus* aqueous system M1 (34.6 kJ mol^−1^), and a 594.7% reduction *versus* propylene glycol (PG) system M3 (198.1 kJ mol^−1^, Fig. 5d and e). This decreased energy barrier correlates with enhanced AzA permeation efficiency in THEDES environments. Component distribution analysis (Fig. 5) demonstrates distinct spatial organization (*z*-direction) in M2: CER3, CHL1 and LAU accumulate in bilayer mid-zones, while DP preferentially localize at interfacial regions. Therefore, it can be hypothesized that the observed THEDES-mediated permeability enhancement is primarily attributed to DP potentially acting as a mediator: (1) improving AzA-lipid compatibility through molecular interactions, and (2) reducing interfacial resistance *via* THEDES-lipid layer associations, thereby facilitating supramolecular AzA transport. To validate the hypothesis, we analyzed the interactions among AzA, DP, and the skin model system. As shown in Fig. 5f, the lipid matrix exhibited weaker interaction energy with AzA in M2 (−10.7 kJ mol^−1^) compared to M1 (−26.6 kJ mol^−1^) and M3 (−46.8 kJ mol^−1^), suggesting reduced transdermal resistance in the M2 system. This conclusion is further supported by two key observations: (1) the weaker AzA-DP interaction energy (−8.8 kJ mol^−1^) and (2) the significantly stronger DP-skin interaction (−522.5 kJ mol^−1^), which collectively explain the reduced AzA permeation barrier observed in Fig. 5e. Finally, total free energy calculations also confirmed both system stability and the presence of weak AzA-DP interactions.

### Study of antibacterial activity

A significant increase in the proliferation of *Propionibacterium acnes* is a major cause one of the important causes of several skin diseases, such as acne. AzA has demonstrated effective bacteriostatic properties, and its antimicrobial activity is crucial for assessing the effectiveness of the THEDES (AzA-DP-4). We assessed its bacteriostatic effect through experiments such as the agar diffusion assay, minimum inhibitory concentration (MIC) test, and minimum bactericidal concentration (MBC) test.^31,32^

**Fig. 6:**
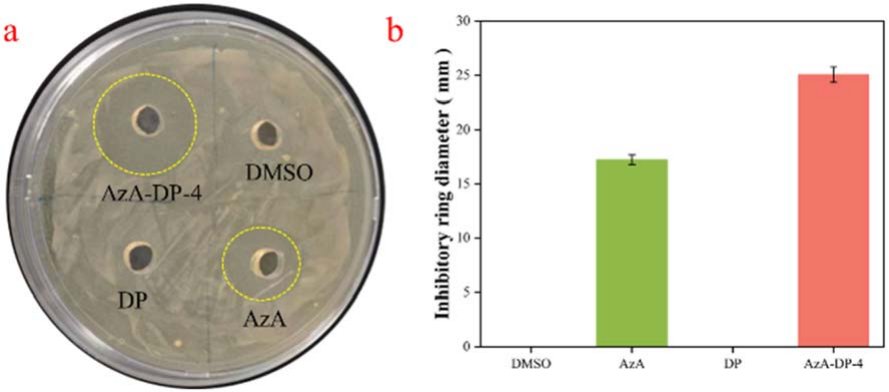
Inhibition ring test (a) inhibition ring; (b) antibacterial circle diameter.

The results of the agar diffusion assay tests are shown in Fig. 6a. DP and solvent control did not exhibit inhibitory properties. AzA-DP-4 and AzA showed significant inhibitory effects on *Propionibacterium acnes*. Concretely, AzA-DP-4 exhibited an inhibitory ring diameter of 25.06 ± 0.71 mm, larger than that of AzA with the same content (17.23 ± 0.45 mm), thereby indicating that the inhibitory characteristics were improved through the formation of THEDES. The minimum inhibitory concentration (MIC) and minimum bactericidal concentration (MBC) of the THEDES (AzA-DP-4) system were 3.13 mg mL^−1^ and 6.25 mg mL^−1^ at the AzA content of 0.98 mg mL^−1^ and 1.96 mg mL^−1^, respectively. At the same level of AzA content, the MIC and MBC of AzA raw material were 1.96 mg mL^−1^ and 3.93 mg mL^−1^, respectively, proving that the THEDES (AzA-DP-4) system had better antibacterial performance.

### Analysis of anti-inflammatory efficacy

**Fig. 7:**
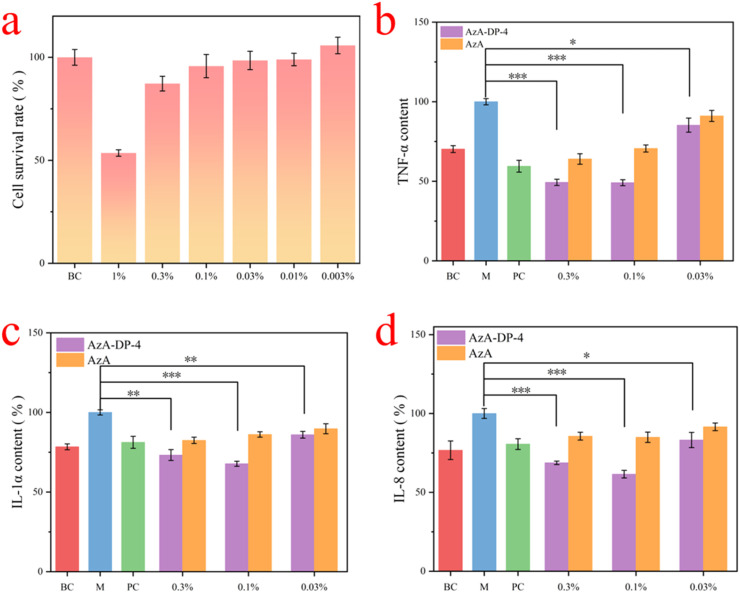
Anti-inflammatory efficacy (a) cell activity test; effect on (b) TNF-α; (c) IL-1α; (d) IL-8.

The anti-inflammatory efficacy of AzA-DP-4 was investigated using HaCaT keratin-forming cells. To determine the appropriate dosage, we evaluated the effect of different concentrations of AzA-DP-4 on the metabolic activity of HaCat cells. The experimental results are shown in Fig. 7a. At a concentration of 1%, there was a notable decrease in cell activity. In the concentration range of 0.3% to 0.003%, the cell survival rate exceeded 85%. Thus, the tested concentrations of the THEDES system were selected as 0.3%, 0.1% and 0.03%, respectively.

The anti-inflammatory activity was assessed by three common pro-inflammatory cytokines (TNF-α, IL-1α, and IL-8). As shown in Fig. 7b–d, both AzA-DP-4 and AzA reduced the secretion of these three inflammatory factors compared to the model group. In comparison to AzA, AzA-DP-4 exhibited markedly stronger inhibitory effects on these three inflammatory factors at the same meter mass concentration of AzA. The inhibitory effect of AzA-DP-4 on TNF-α, IL-1α, and IL-8 was most significant at a concentration of 0.1%, demonstrating inhibition rates of 50.66%, 32.23%, and 36.69%, respectively. These results show that the AzA synergizes with DP, further amplifying the overall anti-inflammatory effect of the THEDES.

### Clinical efficacy test

Although AzA is widely used to clinically treat acne-related disorders, it is not applicable to sensitive skin. The above results show that AzA-DP-4 has higher anti-inflammatory activities and better transdermal efficiency, while also exhibiting low toxicity and irritation. Therefore, AzA-DP-4 is an excellent candidate for the universal treatment of acne. Pre-clinical skin irritation tests were performed to ensure the clinical safety of THEDES, and the results of the tests are shown in Table S3, where none of the 20 volunteers showed any adverse symptoms such as redness, swelling, peeling, and allergic reactions after 2 days, and were ready to proceed to the subsequent clinical trial.

**Fig. 8:**
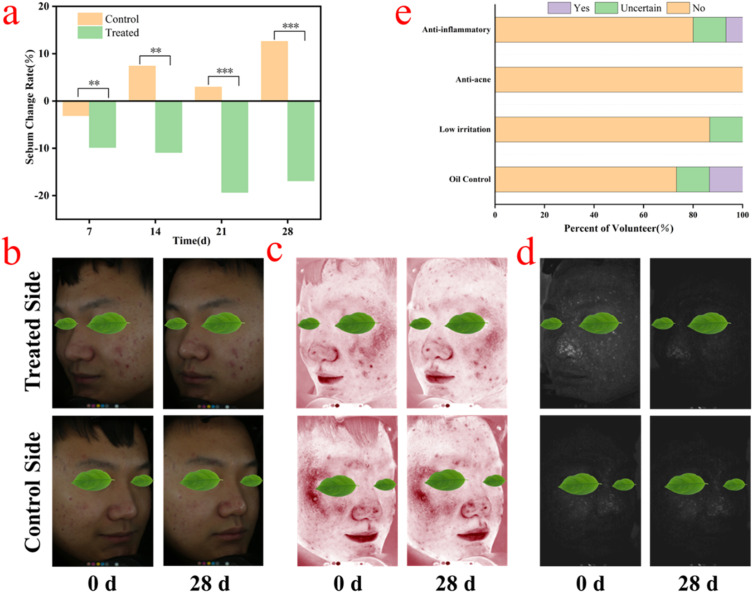
Clinical trial test results of AzA-DP-4 (a) change rate of sebum on the skin surface of volunteers after 28 days (**p < 0.01, ***p < 0.001); (b) a representative volunteer's survey questionnaire on changes in papules; (c) red area; (d) porphyrin area before and after treatment; (e) post clinical trial volunteer survey.

Excessive secretion of sebaceous glands is an important factor in triggering acne. Patients who secrete too much sebum tend to clog their pores, leading to the formation of pimples and nodules, and further leading to acne lesions. Therefore, the skin surface sebum level is an important indicator for assessing the occurrence of acne.^33^ The rate of change of skin surface sebum of the volunteers before and after the clinical trial is shown in Fig. 8a. Compared with the control group, the level of skin surface sebum was reduced by 16.89% after 28 days of treatment with AzA-DP-4, which demonstrated a good oil-control ability to effectively reduce the excessive secretion of sebaceous glands. In addition, AzA-DP-4 was effective in relieving acne and improving skin red zone lesions, as shown in Fig. 8b–d. Porphyrin is a metabolite of some bacteria that parasitize hair follicles. During the development of acne, the increased secretion of sebaceous glands leads to the accumulation of porphyrin, and the degree of inflammatory reaction on the skin surface can be determined by testing the amount of porphyrin content on the skin surface. Red zones indicate skin problems related to blood vessels, such as acne, inflammation, skin sensitivity, and hemangiomas, *etc.* After 28 days, the volunteers' facial acne condition remained basically unchanged or even worsened in the control group, while the acne condition of the experimental group treated by AzA-DP-4 improved with the area of porphyrins and the area of red zones reduced by 12.3%^34^ and 17.5%, respectively. This is a notable improvement of the surface inflammatory reaction of the skin.

In addition, a questionnaire survey was conducted on the volunteers after the follow-up test cycle, and the reported results are shown in Fig. 8e. All the volunteers believed that AzA-DP-4 had an acne-eliminating effect, and most of the volunteers indicated that AzA-DP-4 had an anti-inflammatory and oil-controlling effect, and more than 80% of the volunteers believed that AzA-DP-4 had a low irritation and a mild effect, demonstrating a good safety of use. In conclusion, AzA-DP-4 showed excellent acne efficacy in clinical treatment.

### Acne mechanism of action analysis

**Fig. 9:**
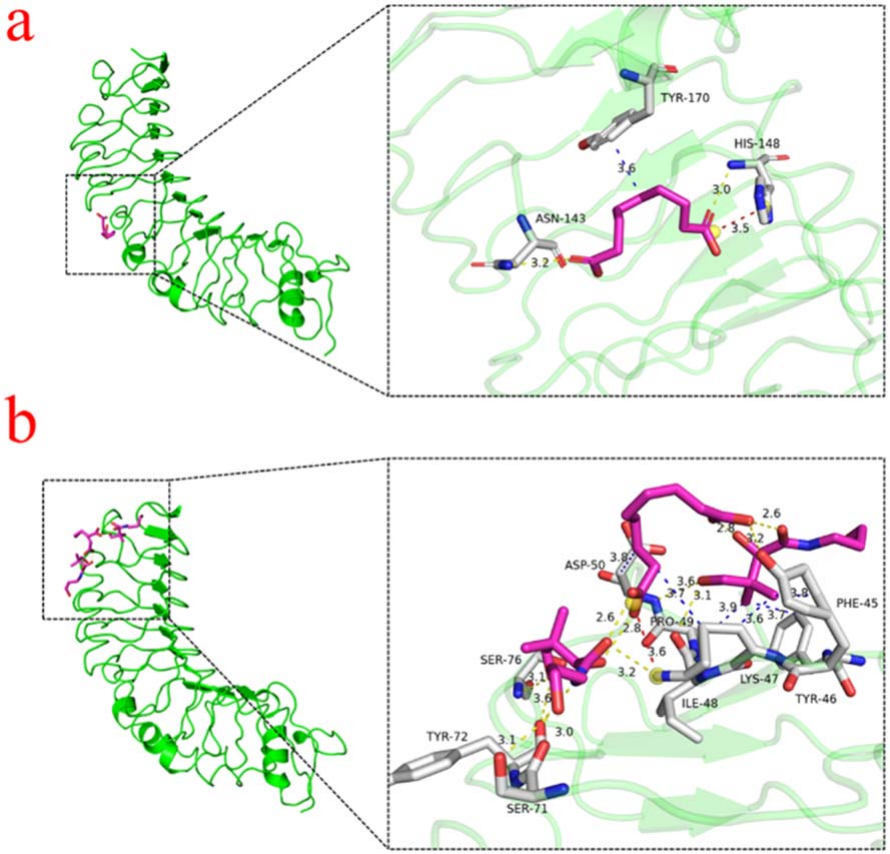
Molecular docking patterns of (a) AzA; (b) AzA-DP-4 with TLR4.

In the treatment of acne, AzA reduces the inflammatory response by decreasing the expression of TLR4 on the surface of keratinocytes and inhibiting its activation of the NF-κB pathway.^35^ This mechanism provides a theoretical basis for examining the mechanisms involved in the treatment of acne using AzA. It not only helps to alleviate skin inflammation, but also promotes the healing of acne by regulating the skin's immune response.^36^ Molecular docking allowed us to analyze the intermolecular interaction patterns of AzA and AzA-DP-4 with the TLR4 protein receptor, thereby elucidating the mechanism of action in the treatment of acne. As shown in Fig. 9, the binding energies of AzA and AzA-DP-4 to TLR4 were observed to be −4.4 kcal mol^−1^ and −5.7 kcal mol^−1^, respectively.

This suggests AzA-DP-4 exhibits stronger binding activity with TLR4, which may be attributed to the more stable hydrogen bonding network in THEDES. In addition, the docking sites of AzA and AzA-DP-4 with TLR4 are presented in Tables S4 and S5. In comparison to AzA, AzA-DP-4 and TLR4 exhibited some new binding sites (such as 46A-TYR, 47A-LYS, 48A-ILE, 50A-ASP, 71A-SER, 72A-TYR, 76A-SER, 45A-PHE, 46A-TYR, 47A-LYS, 49A-PRO, 50A-ASP, 47A-LYS) along with an increased number of hydrogen bonds or other interaction forces. These new sites and stronger binding activity with TLR4 results in synergistic effects and enhancing clinical therapeutic efficacy of AzA-DP-4 in acne, corresponding to the anti-inflammatory efficacy results.

## Conclusions

In this study, an AzA-DP-THEDES system was successfully developed to effectively address the permeability and water solubility issues of AzA. DP was chosen as the ligand, and the optimal molar ratio of AzA : DP was determined to be 1 : 2 using POM test and theoretical calculations. Furthermore, it was demonstrated that the THEDES was formed through intermolecular hydrogen bonding by FTIR spectroscopy, 1H NMR, and TGA. In this study, an azelaic acid-D-panthenol deep eutectic solvent (DES) was successfully developed. DSC characterization confirmed that all formulations are homogeneous single-phase amorphous undercooled liquids, and systematic thermodynamic modeling verified the significant deviation of the system from ideal mixing behavior, which provides sufficient thermodynamic substantiation for DES formation. The optimal 1 : 2 formulation exhibited the strongest intermolecular hydrogen–bond interaction, which is the intrinsic reason for its excellent solubilization, skin permeation and biological activity performance. The AzA-DP-4 system exhibited excellent water solubility, stability and transdermal permeability after being diluted to different concentrations. It also exhibited low irritation and toxicity to the skin, making it safe for application. Additionally, it demonstrated high bioactivity, showcasing remarkable antibacterial properties against Propionibacterium acnes, as well as anti-inflammatory effects on TNF-α, IL-1α, and IL-8. The system has also proven effective in eliminating acne and exhibiting anti-acne efficacy in clinical trials. The mechanisms of action in the treatment of acne and skin permeation are finally investigated through molecular docking analysis and molecular dynamics simulation, suggesting that AzA-DP THEDES can be an effective candidate to treat acne as TDDS formulation.

## Experiments

### Material preparation

Azelaic acid (pharmaceutical grade ≥99%) was obtained from Jiangsu Xuansen Pharmaceutical Co., Ltd (Jiangsu, China); D-panthenol (analytical grade 99.7%, AR) and agar powder from Shanghai Haohong Biomedical Technology Co., Ltd (Shanghai, China); and Reinforced *Clostridium difficile* Medium (RCM) from Qingdao Haibo Biotechnology Co., Ltd. Propionibacterium acnes (BNCC 330605) and HaCaT cells were sourced from Beijing Beinan Chuanglian Biotechnology Co., Ltd and Wuhan Baijiekang Biotechnology Co., Ltd, respectively. ELISA kits for IL-1α, TNF-α, and IL-8 were procured from Abcam (Shanghai, China). All other chemicals, of chromatographic grade, were purchased from Sinopharm Chemical Reagent Co., Ltd (Shanghai, China).

### Preparation of THEDES

AzA and DP were mixed at different molar ratios (3 : 1, 2 : 1, 1 : 1, 1 : 2, 1 : 3) and added to a tightly sealed round-bottom flask. The mixture was stirred continuously at 90 °C under magnetic stirring for 5 h until a homogeneous and transparent liquid was formed, before being cooled to room temperature for subsequent characterization tests.

### Polarized light microscopy (POM) test

The phase behavior of AzA-DP-3, 4, and 5 systems maintaining homogeneous transparency after 24-hours equilibration was investigated using polarized optical microscopy (POM). Samples were analyzed at 25 °C by uniformly depositing a thin layer of THEDES on microscope slides. Optical characterization was performed at 5×, 10×, and 20× magnifications to identify distinct phase transitions, including solid, semi-solid, crystalline, and liquid states across the three systems.

### Water solubility and stability test

The AzA-DP-4 system containing 31.4 wt% AzA was sequentially diluted with deionized water to achieve AzA concentrations of 1, 3, 5, 10, and 20 wt%. All prepared solutions were stored in sealed vials under ambient conditions (25 °C) for 30 days, with systematic monitoring of phase behavior and stability through daily visual inspection. The physical stability of AzA-DP-4 against recrystallization was evaluated under accelerated conditions. Aqueous solutions of AzA-DP-4 containing 1%, 5%, 10%, and 20% (w/w) AzA were prepared and sealed in 5 mL glass vials. The vials were placed in a constant temperature chamber maintained at 45 ± 2 °C for 30 days. At regular intervals (daily for the first week, and then weekly), the samples were visually examined against a black-and-white background for any changes in appearance, including turbidity, precipitation, or phase separation. Photographs were taken at the beginning and at the end of the study period.^13^C NMR spectra were recorded on a [Bruker AVANCE III 400 MHz] nuclear magnetic resonance spectrometer at 25 °C, with a scanning frequency of 100 MHz. The samples were dissolved in deuterated dimethyl sulfoxide (DMSO-*d*_6_) with a concentration of ∼50 mg mL^−1^, and tetramethylsilane (TMS) was used as the internal standard for chemical shift calibration. The number of scans was set to 2048 to ensure sufficient signal-to-noise ratio.

### Molecular simulation calculations

All calculations were performed using Gaussian 16 with the M06-2X functional. Geometry optimizations and vibrational frequency analyses (confirming local minima) utilized the 6-31G (d,p) basis set, while single-point energy calculations employed the 6-311 + G (d,p) basis set. Noncovalent interactions, electrostatic potentials, and electron topology features across DP-AzA molar ratios were systematically investigated through Multiwfn (v3.8, Tian Lu) for AIM, NCI, and ESP analyses, with visualization implemented in VMD.

### Characterization

The chemical structures of AzA, DP, and AzA-DP-4 were characterized using FT-IR (Bio-Rad FTS6000, 4000–500 cm^−1^, 4 cm^−1^ resolution), ^1^H NMR (Bruker AVANCE III HD 400 MHz, DMSO-*d*_6_ solutions, 10 mg/0.6 mL), and thermogravimetric analysis (Mettler TGA2 SF/1100, 10 °C min^−1^ under N_2_ from 30–550 °C, 50 mL min^−1^ purge flow). All thermal and spectroscopic measurements were conducted in triplicate to ensure reproducibility.DSC tests were performed using a NETZSCH DSC 204 F1 Phoenix differential scanning calorimeter under a high-purity nitrogen atmosphere (50 mL min^−1^). 5–8 mg of each sample was accurately weighed into a sealed aluminum crucible with a micro pinhole, with an empty sealed aluminum crucible as reference. The temperature program was: equilibrate at 50 °C for 3 min to eliminate thermal history, cool to −100 °C at 10 °C min^−1^ and equilibrate for 5 min, then heat to 50 °C at 10 °C min^−1^. The second heating curve was collected for analysis, and the midpoint temperature of the glass transition step was taken as the *T*_g_ value.

### Karl Fischer titration

The moisture content of the prepared DES formulations was determined by a [Mettler Toledo EVA V1] Karl Fischer titrator at 25 °C. Each sample was tested in triplicate, and the average value was taken as the final moisture content.

### Thermodynamic modeling and calculation

The thermodynamic properties of the amorphous AzA-DP DES system were characterized and modeled *via* the following methods, which are standard and widely recognized for binary amorphous mixtures:

Ideal mixing *T*_g_ calculation (Fox equation): The ideal mixing *T*_g_ of the binary system was calculated *via* the Fox equation, which assumes no specific intermolecular interaction between components (ideal physical mixing):
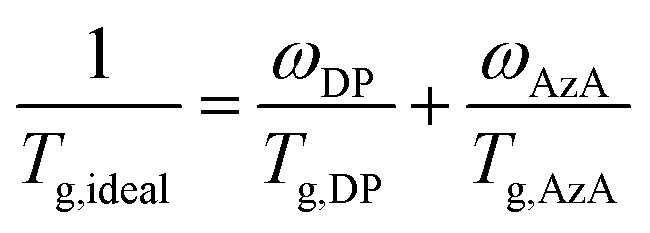
where *T*_g,ideal_ is the ideal mixing *T*_g_ (K), *ω*_DP_ and *ω*_AzA_ are the mass fractions of D-panthenol (DP) and azelaic acid (AzA), *T*_g,DP_ = 240.98 K is the experimentally measured *T*_g_ of pure DP, and *T*_g,AzA_ = 206.5 K is the fitted *T*_g_ of amorphous AzA from the Gordon–Taylor model.

Non-ideal mixing thermodynamic modeling (Gordon–Taylor model): The composition-dependent *T*_g_ data was fitted *via* the Gordon–Taylor model, the standard thermodynamic model for binary amorphous systems, to quantitatively characterize the intermolecular interaction between AzA and DP:
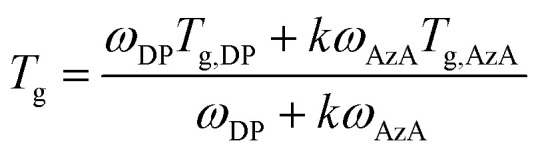
where *T*_g_ is the experimentally measured *T*_g_ of the mixture (K), and *k* is the interaction parameter (deviation from *k* = 1 indicates non-ideal mixing).

Flory–Huggins interaction parameter and activity coefficient calculation: the Flory–Huggins solution theory was used to calculate the intermolecular interaction parameter *χ* and the activity coefficient of AzA, which is the standard method for amorphous binary systems without measurable fusion enthalpy (Δ*H*_fus_). The activity coefficient of AzA (solute, component 2) was calculated *via* the classic formula:
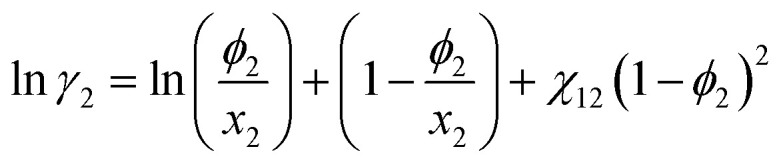
where *γ*_2_ is the activity coefficient of AzA, *x*_2_ and *ϕ*_2_ are the molar fraction and volume fraction of AzA, respectively, and *χ*_12_ is the Flory–Huggins interaction parameter derived from the Gordon–Taylor *k* value *via* the empirical correlation widely verified in DES and co-amorphous systems.

### Toxicity tests

#### Percutaneous toxicity test

The experimental animals were 10 SPF-grade Sprague-Dawley (SD) rats (5 males and 5 females), weighing 230–250 g. The rats were quarantined and qualified for the experiment. Using a single-dose transdermal toxicity test, AzA-DP-4 was evenly applied to the dorsal skin of the rats at a dose of 2180 mg kg^−1^ body weight. The application site was then covered with a thin film and fixed with non-irritating adhesive tape to prevent the rats from licking or ingesting the sample. The occlusive contact was maintained for 24 h. At the end of the exposure period, the residual sample was gently removed using warm water. The rats were observed for 14 days to monitor signs of poisoning and mortality. At the end of the observation period, all rats were subjected to gross necropsy to examine for abnormal changes in major organs.

#### Irritation test

##### Dermal irritation test

Four New Zealand rabbits (2–2.5 kg) were clipped to expose bilateral dorsal skin (3 × 3 cm per area). AzA-DP-4 (0.5 mL) was applied occlusively (gauze/cellophane bandaging) to one site daily for 14 days; contralateral sites served as untreated controls. Prior to each application, residual material was removed with purified water and regrown hair reclipped. Reactions were scored at 1 h post-application according to China's 2015 Cosmetic Safety Standard criteria.

##### Acute ocular irritation test

Three rabbits underwent pre-test ocular screening (fluorescein sodium staining). AzA-DP-4 (0.1 mL) was instilled into one conjunctival sac per rabbit with brief lid closure; contralateral eyes were controls. Test eyes remained unrinsed for 24 h. Observations (cornea/iris/conjunctiva) were recorded at 1, 24, 48, 72 h, and Days 4/7 using the 2015 Cosmetic Standard grading system. If irritation persisted at Day 7, observations extended to Day 21 with fluorescein staining at terminal endpoints. All animal experimental procedures in this study were reviewed and approved by the Ethics Committee of Hunan Shanshui Testing Co., Ltd (Approval No. JY20240096). All experiments were strictly performed in compliance with the ARRIVE 2.0 guidelines, as well as national and institutional regulations for the care and use of laboratory animals. All efforts were made to minimize animal suffering during the experiment, and the number of experimental animals was optimized on the premise of ensuring the statistical validity of the results.

##### *In vitro* permeability test

Transdermal permeation of AzA-DP-4 was evaluated using Franz diffusion cells mounted with intact porcine skin (stratum corneum facing diffusion chamber). Test formulations included AzA-DP-4 at 1%, 3%, 5% and 10% w/w in propylene glycol and a 1% w/w AzA control. The receiving chamber contained degassed PBS (pH 7.4, 0.01 M) maintained at 37 ± 0.1 °C with continuous stirring (350 rpm). Subsequently, 2 mL of each formulation was added to the diffusion chamber. Aliquots (1 mL) were collected from the receiving chamber at 2, 4, 6, 12, and 24 h, with immediate replenishment of fresh medium to maintain sink conditions.

The samples collected from the transdermal permeation experiment were filtered through 0.45 µm microfiltration membranes. The concentration of azelaic acid in the filtrates was determined *via* high-performance liquid chromatography (HPLC) A C18 column (4.6 mm × 250 mm, 5 µm) was used. The mobile phase consisted of 60% methanol and 40% 0.1% phosphoric acid aqueous solution. The column temperature was maintained at 30 °C, with a flow rate of 1.0 mL min^−1^. The detection wavelength was set at 210 nm, and the injection volume was 10 µL, and the results were recorded. Subsequently, the cumulative permeation amount (1) and permeation rate (2) of the drug (azelaic acid) were calculated using the following formula:1
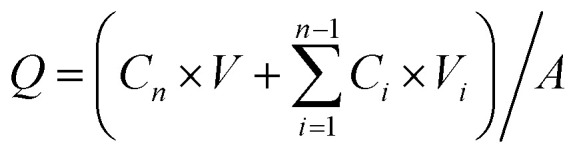
2
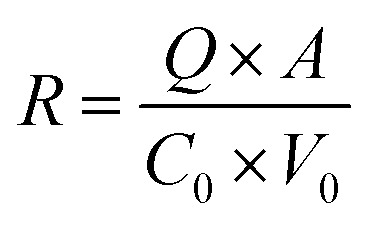
where *Q* is the cumulative transdermal permeation of drug per unit area (µg cm^−2^); *V* is the volume of the receiving cell (7 mL); *A* is the area of diffusion (3 cm^2^); *C*_*n*_ denotes the concentration of the drug measured at the *n*th sampling point (µg mL^−1^); and *V*_i_ is the volume of sampling (1 mL). *R* is the drug permeation rate (%); *C*_0_ is the initial concentration of the drug (µg mL^−1^); and *V*_0_ is the initial added volume of the drug (2 mL).

Post-permeation skin samples were rinsed with PBS, fixed in 4% paraformaldehyde, and processed for histopathological (H&E-stained paraffin sections) and fluorescence analyses. For permeation pathway tracking, FITC (0.1% w/v) was incorporated into test systems prior to diffusion studies. Cryosectioned vertical skin slices (10 µm) underwent dual-mode imaging: confocal laser scanning microscopy (CLSM) and epifluorescence microscopy (*λ*_ex_ 488 nm/*λ*_em_ 520 nm) to spatially resolve fluorophore distribution across skin strata.

#### Molecular dynamics permeation simulation

Simulation of transdermal mechanism by computational methods: transdermal simulation. MD simulations were conducted using the GROMACS software package (version 2018.8).^37^ The CHARMM 36M force field was used for all potential function terms to calculate interatomic interactions.^38^ The total potential energy is expressed as a combination of valence terms, including bond stretching, angle bending, torsion, and non-bonded interactions. The non-bonded interactions between atoms were described by the Lennard-Jones potential, and standard geometric mean combination rules were used for the van der Waals interactions between different atom species. In the simulation, the systems were initialized by minimizing the energies of the initial configurations using the steepest descent method. Subsequently, a 100 ps NVT and NPT simulation was conducted to achieve equilibrium. Subsequently, AzA molecules were extracted from pure water (M1), and DP aqueous solutions (M2), and PG solutions (M3) through the skin membrane. A spring coefficient of 1000 kJ mol^−1^ nm^−2^ and a pull speed of 0.1 nm ns^−1^ were selected. A weighted histogram analysis method was used to extract the PMF. In all simulations, the temperature was maintained at 303.15 K using the v-rescale thermostat algorithm.^39^ The bond lengths were constrained using the LINCS algorithm and periodic boundary conditions were applied in all directions.^40^ Short-range non-bonded interactions were terminated at a distance of 1.2 nm, and long-range electrostatic interactions were calculated using the particle mesh Ewald method.^41^ The trajectories were stored at 100 ps intervals and visualized using VMD 1.9.3.

#### Bacteriostatic testing

Antimicrobial efficacy of AzA, DP, and AzA-DP-4 against *Cutibacterium acnes* (formerly *Propionibacterium acnes*) was assessed *via* agar diffusion assays complemented by MIC (the minimum inhibitory concentration)/MBC (the minimum bactericidal concentration) determinations. Bacterial inocula (1 × 10^6^ CFU mL^−1^) were prepared from anaerobic cultures grown in reinforced clostridial medium (RCM) at 37 °C for 48 h.

Antimicrobial activity was evaluated using the quadrant-partitioned RCM agar plates inoculated with *Cutibacterium acnes* (1 × 10^6^ CFU mL^−1^). AzA-DP-4 (100 mg mL^−1^ in DMSO), along with AzA (31.4 mg mL^−1^) and DP (68.6 mg mL^−1^) at THEDES-equivalent ratios, were loaded into aseptically punched wells (100 µL per well), with DMSO serving as a vehicle control. Inhibition zone diameters were quantified after 48 h anaerobic incubation (37 °C). Triplicate experiments provided mean inhibitory measurements.

MIC and MBC values for AzA-DP-4 and its components (AzA, DP) were determined *via* microdilution in 96-well plates. Samples were serially diluted (200–0.78 mg mL^−1^ for AzA-DP-4; THEDES-equivalent concentrations for AzA/DP) in reinforced clostridial medium (RCM). Each well received 10 µL of Cutibacterium acnes inoculum (1 × 10^6^ CFU mL^−1^), except controls: growth control (medium + bacteria), solvent control (DMSO), and blank (medium only). MIC was recorded as the lowest concentration showing no visible growth after 48 h anaerobic incubation (37 °C). MBC was determined by subculturing clear wells onto fresh RCM agar and defined as the lowest concentration achieving ≥99.9% kill rate. Tests were performed in triplicate.

#### Anti-inflammatory efficacy test

HaCaT cell viability was assessed *via* MTT assay in 96-well plates. Following overnight incubation (37 °C, 5% CO_2_), cells were exposed to six AzA-DP-4 concentrations (six replicates each) alongside controls: zeroing (medium only), blank control (untreated cells with FBS-supplemented medium), and untreated cells. After 24 h incubation, supernatants were replaced with MTT-containing medium (3 h, dark). Formazan crystals were dissolved in DMSO (100 µL per well), and absorbance was measured at 490 nm Viability was calculated as the following formula (3):3
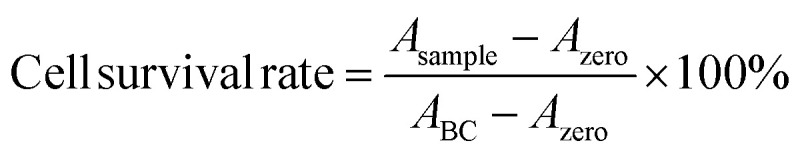


HaCaT cells were seeded in 12-well plates and incubated overnight (37 °C, 5% CO_2_) prior to inflammatory factor analysis.

Experimental groups included: blank control (BC): culture medium only, model control (M): medium + LPS (1 µg mL^−1^), positive control (PC): medium + LPS (1 µg mL^−1^) + dexamethasone (20 µM), test groups: medium + LPS (1 µg mL^−1^) + AzA-DP-4 or AzA.

After 24 h incubation, supernatants were collected, stored at −80 °C, and analyzed for TNF-α, IL-1α, and IL-8 levels using commercial ELISA kits according to manufacturer protocols. Inflammatory factor expression was calculated relative to the M group.

#### Clinical efficacy tests

##### Pre-clinical skin irritation test

We conducted a clinical skin irritation assessment in 20 healthy volunteers (21–40 years) using 3% w/w AzA aqueous solution (diluted from AzA-DP-4). Test sites (inner forearms) received 0.25–0.5 mL samples under 24 h occlusion. Following removal, cutaneous reactions were assessed by visual scoring at 0.5, 24, and 48 h post-removal.

##### Anti-acne clinical efficacy test

An anti-acne efficacy study enrolled 15 volunteers (18–30 years, acne-afflicted, no comorbidities). Subjects abstained from comedolytic agents, acne cosmetics, or oral anti-inflammatories for ≥14 days pre-test and throughout the 21-day trial. Test areas received topical application of diluted AzA-DP-4 aqueous formulation (3% w/w AzA) twice daily; contralateral sites served as untreated controls. While mild cleansers were permitted, all other topical agents were prohibited.

Outcome assessments at weekly intervals included: Sebumetry (Sebumeter®), quantification of acne lesions, porphyrins, and erythema using VISIA-CR® facial imaging.

#### Mechanisms of action studies for acne treatment

Molecular docking simulations of AzA-DP-4 and AzA with TLR4 (PDB:3UL7) were performed using AutoDock Vina 1.1.2. The receptor structure was prepared in PyMOL 2.4 (removal of water/ligands; hydrogen atom addition), while ligand geometries were energy-minimized (ChemDraw 20.0). PDBQT file conversion employed AutoDock Tools 1.5.6. A grid box encompassing the entire protein defined the search space. Semi-flexible docking generated 9 binding poses per ligand, with the global minimum energy conformation (lowest binding energy + highest cluster frequency) identified as the optimal binding mode. Protein–ligand interactions were visualized using PyMOL 2.4 and PLIP.

## Ethical statement

All experiments involving human volunteers were performed in accordance with the guidelines of the World Medical Association Declaration of Helsinki and the Ethical Review Measures for Biomedical Research Involving Human Beings of the People's Republic of China. The study protocol was approved by the Biomedical Ethics Committee of Jiangnan University. Written informed consent was obtained from all human participants prior to their enrolment in the study.

## Conflicts of interest

There are no conflicts to declare.

## Supplementary Material

RA-016-D5RA09988A-s001

## Data Availability

The data that support the findings of this study are available in the supplementary information (SI) of this article. Supplementary information: acceleration stability test graph, DTG data, DSC data, nuclear magnetic resonance carbon spectrum data, skin irritation test results, eye irritation test results, molecular docking sites, *etc*. See DOI: https://doi.org/10.1039/d5ra09988a.
