# Supplementary material for: Azelaic acid-integrated therapeutic deep eutectic systems: overcoming solubility and permeability barriers for enhanced transdermal drug delivery
Source: RSC Adv. 2026 Apr 13;16(21):19270–84. doi: 10.1039/d5ra09988a (PMC13071801; doi:10.1039/d5ra09988a)
Supplement: RA-016-D5RA09988A-s001 [file RA-016-D5RA09988A-s001.pdf]

Supplementary Information (SI) for RSC Advances.
This journal is © The Royal Society of Chemistry 2026

Azelaic Acid-Integrated Therapeutic Deep Eutectic Systems: Overcoming Solubility and Permeability Barriers for Enhanced Transdermal Drug Delivery

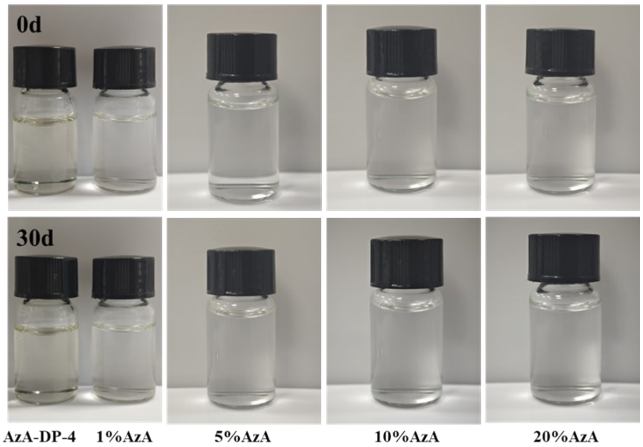

S1 stability of AzA-DP-4.

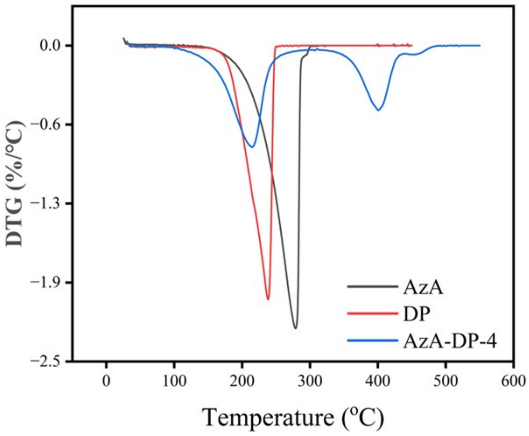

S2 Characterization of AzA, DP and THEDES DTG

S3 Key thermal degradation parameters of AzA, DP and AzA-DP-4 system

Sample	Degradation Stage	Δm /%	T_{onset} /°C	T_{max} /°C	T_{end} /°C
DP	Stage 1	99.8	130	230	280
AzA	Stage 1	99.7	250	280	350
AzA-DP-4 THEDES	Stage 1 (DP-dominated)	68.6	132	220	300
AzA-DP-4 THEDES	Stage 2 (AzA-dominated)	31.4	300	390	480

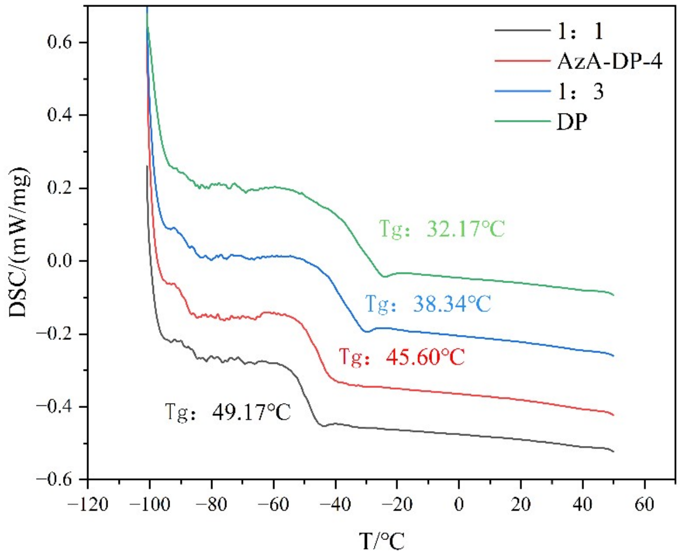

S4 Characterization of 1：1, AzA-DP-4, 1:3 and DP DSC

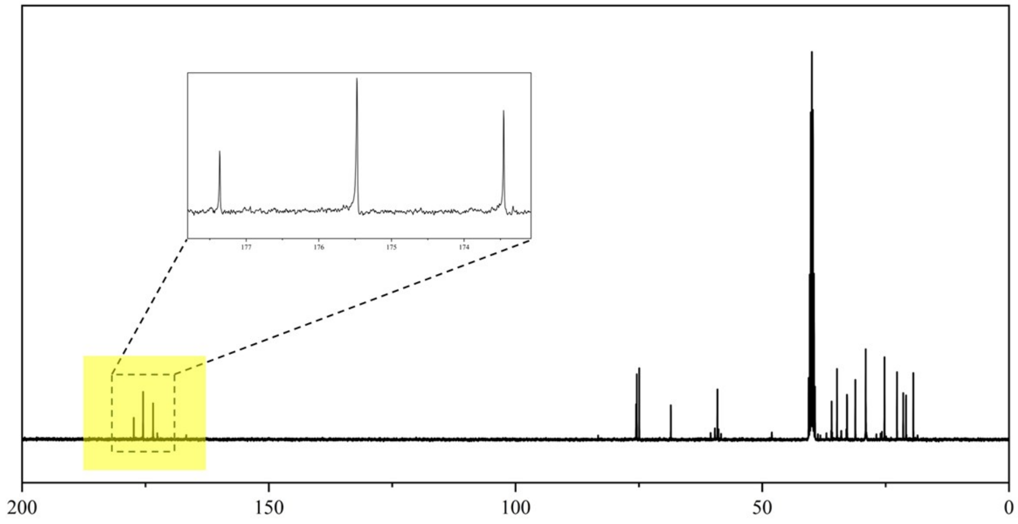

S5 The ^{13}C NMR of AzA-DP-4

S6 Comparison of measured Tg and ideal mixing Tg of AzA-DP systems

AzA:DP molar ratio	Measured Tg (℃)	Measured Tg (K)	Ideal Tg (Fox equation, K)	Deviation (K, Measured - Ideal)
0:1 (pure DP)	-32.17	240.98	240.98	0.00
1:3	-38.34	234.81	233.12	+1.69
1:2 (optimal ratio)	-45.60	227.55	228.90	-1.35

AzA:DP molar ratio	Measured Tg (℃)	Measured Tg (K)	Ideal Tg (Fox equation, K)	Deviation (K, Measured - Ideal)
1:1	-49.17	223.98	224.87	-0.89

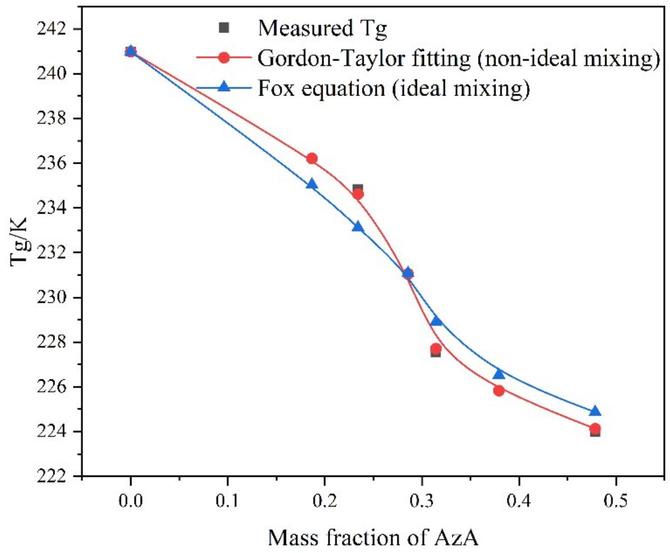

S7 Composition-dependent Tg profile of AzA-DP systems: measured Tg (black), ideal mixing line (Fox equation, blue), and Gordon-Taylor fitting curve (red).

S8. Core parameters of Gordon-Taylor model fitting

Parameter	Value	Physical Meaning
Fixed Tg,DP	240.98 K (-32.17℃)	Experimentally measured Tg of pure DP
Fitted Tg,AzA	206.5 K (-66.65℃)	Tg of amorphous AzA obtained via fitting
Interaction parameter k	0.72	Significant deviation from 1 (ideal mixing), confirming strong non-ideal intermolecular interaction
Goodness of fit R^2	0.9992	Excellent fitting effect, reliable modeling results

S9. Results of skin irritation test

Application	Number	Skin irritation reaction score

Number of days	of animals (piece)	Sample group			Control group		
		erythema	edema	Total score	erythema	edema	Total score
1	4	0	0	0	0	0	0
2	4	0	0	0	0	0	0
3	4	0	0	0	0	0	0
4	4	0	0	0	0	0	0
5	4	0	0	0	0	0	0
6	4	0	0	0	0	0	0
7	4	0	0	0	0	0	0
8	4	0	0	0	0	0	0
9	4	0	0	0	0	0	0
10	4	0	0	0	0	0	0
11	4	0	0	0	0	0	0
12	4	0	0	0	0	0	0
13	4	0	0	0	0	0	0
14	4	0	0	0	0	0	0
Average points per animal over 14 days		0	0	0	0	0	0
Average points per animal per day		0	0	0	0	0	0

S10. Results of Acute Eye Irritation Test

Animal Number	part	Eye irritation response score							
		1 h		24 h		48 h		72 h	
		sample	control	sample	control	sample	control	sample	control
1	cornea	0	0	0	0	0	0	0	0
	iris	0	0	0	0	0	0	0	0
	conjunctiva	0	0	0	0	0	0	0	0
2	cornea	0	0	0	0	0	0	0	0
	iris	0	0	0	0	0	0	0	0
	conjunctiva	0	0	0	0	0	0	0	0
3	cornea	0	0	0	0	0	0	0	0
	iris	0	0	0	0	0	0	0	0
	conjunctiva	0	0	0	0	0	0	0	0
Integral mean value	cornea	0	0	0	0	0	0	0	0
	iris	0	0	0	0	0	0	0	0
	conjunctiva	0	0	0	0	0	0	0	0

S11. Skin conditions of volunteers in different time periods

Group	Time	The number of volunteers with different skin scores				
		0[a]	1[b]	2[c]	3[d]	4[e]
Control	0.5 h	20	0	0	0	0
	24 h	20	0	0	0	0
	48 h	20	0	0	0	0
Experiment	0.5 h	20	0	0	0	0
	24 h	20	0	0	0	0
	48 h	20	0	0	0	0

(a) Negative; (b) Suspected reaction, with only faint spots; (c) Weakly positive reaction, with erythema, infiltration, edema, and possibly papules; (d) Strongly positive reaction, with erythema, infiltration, edema, papules, herpes, which may exceed the test area; (e) Strongly positive reaction, with obvious erythema, severe infiltration, edema, confluent measles, exceeding the test area.

S12 Information on the action sites of azelaic acid and TLR4

Types of acting forces	Amino acid site information （residue）	Amino acid name
Hydrogen Bonds	143A	ASN
	148A	HIS
Hydrophobic Interaction	170A	TYR
Salt Bridges	148A	HIS

S13. Information on the interaction sites between NADES (AzA-DP-4) and TLR4

Types of forces	Amino acid site information （residue）	Amino acid name
Hydrogen Bonds	46A	TYR
	47A	LYS
	48A	ILE
	50A	ASP
	71A	SER
	72A	TYR
	76A	SER
Hydrophobic Interaction	45A	PHE
	46A	TYR
	47A	LYS
	49A	PRO
	50A	ASP
Salt Bridges	47A	LYS
